# Hedgehog Signaling Regulates the Survival of Gastric Cancer Cells by Regulating the Expression of Bcl-2

**DOI:** 10.3390/ijms10073033

**Published:** 2009-07-06

**Authors:** Myoung-Eun Han, Young-Suk Lee, Sun-Yong Baek, Bong-Seon Kim, Jae-Bong Kim, Sae-Ock Oh

**Affiliations:** 1 Department of Anatomy, School of Medicine, Pusan National University, Korea; E-Mails: corsaro@paran.com (M.E.H.); 96lys@hanmail.net (Y.S.L.); sybaek@pusan.ac.kr (S.Y.B.); kimbs@pusan.ac.kr (B.S.K.); kimjb@pusan.ac.kr (J.B.K.); 2 Medical Research Center for Ischemic Tissue Regeneration, Pusan National University, Beomeo-Ri, Mulgeum-Eup, Yangsan, 626-770, Korea

**Keywords:** gastric cancer, hedgehog signaling, apoptosis, Bcl-2

## Abstract

Gastric cancer is the second most common cause of cancer deaths worldwide. The underlying molecular mechanisms of its carcinogenesis are relatively poorly characterized. Hedgehog (Hh) signaling, which is critical for development of various organs including the gastrointestinal tract, has been associated with gastric cancer. The present study was undertaken to reveal the underlying mechanism by which Hh signaling controls gastric cancer cell proliferation. Treatment of gastric cancer cells with cyclopamine, a specific inhibitor of Hh signaling pathway, reduced proliferation and induced apoptosis of gastric cancer cells. Cyclopamine treatment induced cytochrome c release from mitochondria and cleavage of caspase 9. Moreover, Bcl-2 expression was significantly reduced by cyclopamine treatment. These results suggest that Hh signaling regulates the survival of gastric cancer cells by regulating the expression of Bcl-2.

## 1. Introduction

Gastric cancer is the second most common cause of cancer deaths worldwide [1] and the 5-year relative survival rate remains poor [2]. Only two survival-influencing factors, the depth of invasion and the presence of regional lymph node involvement, are commonly used in prognosis [3–5]. Compared to other more extensively investigated cancers such as breast, prostate and colon carcinomas, the molecular mechanisms involved in the transformation and progression of gastric cancer are poorly characterized. The histology of gastric carcinomas is conventionally classified into differentiated and undifferentiated types. The intestinal type is a well differentiated tumor, characterized by cohesive neoplastic cells that form gland-like tubular structures, and the diffuse type is a poorly differentiated tumor in which individual cells infiltrate and thicken the stomach wall [2]. The former is frequently accompanied by liver metastasis while the latter is frequently associated with peritoneal dissemination [2].

The Hedgehog (Hh) protein family, which includes Sonic (Shh), Indian (Ihh), and Desert (Dhh) hedgehogs, regulates cell proliferation, tissue generation and cell differentiation during embryogenesis [6–11]. Patched-1 (Ptch-1) serves as the receptor for all of the Hh proteins. In the absence of Hh, Ptch-1 inhibits Smoothened (Smo), a G protein-coupled receptor. When Hh binds to Ptch-1, Smo is disinhibited and initiates a signaling cascade that activates the transcription factor, Gli-1.

The Shh signaling pathway has recently been shown to play a role in the proliferation of a variety of human cancer cells, including basal cell carcinomas(BCCs), medulloblastomas, small cell lung cancer, pancreatic cancer, prostate cancer and gastric cancers. Previous studies have reported that aberrant activation of Shh signaling through Ptch 1 is frequently detected in cases of advanced gastric adenocarcinoma and that this signaling activation is associated with poorly differentiated gastric tumors. Experiments conducted on gastric cancer cells have also shown that cyclopamine, which inhibits cellular responses to Shh signaling by specifically binding to Smo, can inhibit the malignant growth of gastric cancer cells *in vitro* and *in vivo*. Although Hh signaling is critical in the malignant growth of gastric cancer cells, the underlying mechanism is poorly characterized. To reveal the underlying mechanism we used cyclopamine, a specific inhibitor of Smoothened. The present study shows that Hh signaling regulates the survival of gastric cancer cells by regulating the expression of Bcl-2.

## 2. Results

### 2.1. Hh Signaling Regulates the Proliferation of Gastric Cancer Cells

**Figure 1.**
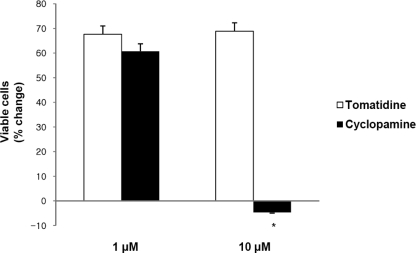
Effects of cyclopamine on proliferation of human gastric cancer cells (SNU16 cells). Proliferation was measured by WST-1. Cells were treated with tomatidine and cyclopamine at the indicated doses for 48 hrs. Data are expressed as percent change (means±SEM) compared to controls. *P<0.001, vs. tomatidine (Student’s two-tailed t-test).

To test whether Hh signaling is involved in the proliferation of gastric cancer cells, we blocked Hh signaling in SNU16 cells gastric cancer cells using cyclopamine, a specific inhibitor of Smo protein. Tomatidine was used as a negative control because it is a structural analogue of cyclopamine. SNU16 cells (1 x 10^5^) were incubated with tomatidine or cyclopamine for 48 hrs at 37°C. As shown in Figure 1, 10 μM of cyclopamine significantly inhibited the proliferation of SNU16 cells, which is consistent with a previous report [6].

### 2.2. Blockade of Hh Signaling Induces Apoptosis of Gastric Cancer Cells

**Figure 2.**
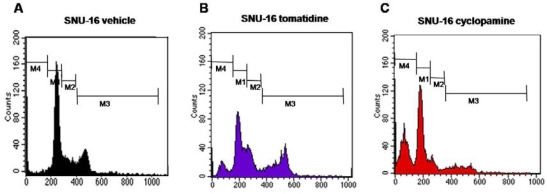

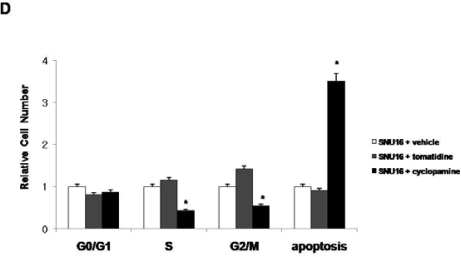
Effects of cyclopamine on cell cycle distribution in SNU16 cells. Flow cytometry was used for analysis of cell cycle in SNU16 cells. Cells were treated with vehicle (A), tomatidine (B) or cyclopamine (C) for 48 hrs before analysis. (D) Quantitation of cell cycle distribution measured in (A), (B) and (C). To compare, the number of cells in vehicle-treated samples was adjusted to 1. The x-axis shows DNA content and the y-axis shows the number of cells. White bar, vehicle; Gray bar, tomatidine; Black bar, cyclopamine. Data are expressed as fold change (means±SEM) and represent four independent experiments. One-way ANOVA revealed significant difference. *P <0.01, vs. tomatidine.

To reveal the mechanism underlying inhibition of proliferation by cyclopamine, we analyzed cell cycle. SNU16 cells (2 × 10^6^) were incubated with vehicle, tomatidine or cyclopamine for 48 hrs at 37°C (Figures 2A, B, and C). Cell cycle (G0/G1, S, G2/M phases) was determined by flow cytometry. As shown in Figure 2, cyclopamine almost arrested the cell cycle of SNU16 cells. Cyclopamine decreased the populations of cells in the S and G2-M phases while hardly affecting those in the G0–G1 phase (Figure 2D). Simultaneously, quantification of apoptotic cells after cyclopamine treatment showed that cyclopamine significantly induced apoptosis of gastric cancer cells (Figure 2D).

### 2.3. Mitochondria are Involved in the Cyclopamine-Induced Apoptosis of Gastric Cancer Cells

**Figure 3.**
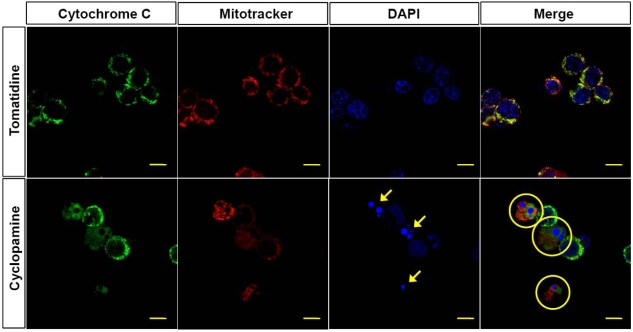
Involvement of mitochondria in cyclopamine-induced apoptosis. Cells treated with 10 μM tomatidine or cyclopamine were fixed with 4% paraformaldehyde for 20 min. Mitotracker Red was used to show mitochondrial morphology. Cytochrome c was detected by anti–cytochrome c antibody and a secondary anti-mouse antibody conjugated to FITC as depicted by green fluorescence. In healthy cells, cytochrome c staining was punctuated as shown for tomatidine-treated cells. In apoptotic cells, cytochrome c release was indicated by diffuse staining, which was found for cyclopamine-treated cells. 4′,6-Diamidino-2-phenylindole (blue fluorescence, arrows) was used as a nuclear counterstain. Data represent 4 independent experiments (scale bar, 5 μm).

To further confirm cyclopamine-induced apoptosis, we stained nuclei with DAPI. We clearly observed fragmented nuclei in the cyclopamine-treated group but not in the tomatidine-treated group (Figure 3). Next, we tested the involvement of mitochondria in cyclopamine-induced apoptosis. We double-stained cells for mitotracker and cytochrome c to reveal the involvement of mitochondria. Confocal microscopy images in Figure 3 show the localization of mitotracker and cytochrome c. Cytochrome c staining was punctuated in tomatidine-treated cells. In cyclopamine-treated cells, cytochrome c release was indicated by diffuse staining.

### 2.4. Hh Signaling Regulates the Expression of Bcl-2

**Figure 4.**
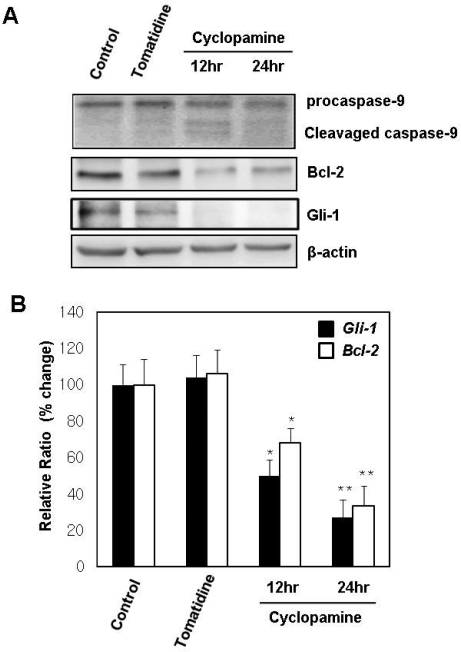
The level of Bcl-2 was reduced by cyclopamine treatment. A. Immunoblot analysis of Bcl-2 and caspase 9 proteins in SNU16 cells treated with tomatidine or cyclopamine (10 μM). Cell lysates (50 μg/lane) from tomatidine or cyclopamine-treated cells were subjected to SDS-PAGE/immunoblotting using antibodies specific for Bcl-2 (Santa Cruz), Caspase 9 (Cell Signaling Technology) and Gli-1 (Santa Cruz). The protein levels of Bcl-2 and Gli-1 were higher in vehicle-treated cells or tomatidine-treated cells than in cyclopamine-treated cells. Western blot analysis was performed with anti-caspase 9 antibody, which detects both pro-caspase 9 protein (47 kDa) and its active form (35 kDa). Activation of caspase 9, as shown by cleavage of pro-caspase 9, was detected in cells treated with cyclopamine. B. The mRNA levels of *Gli-1* and *Bcl-2* were quantified by real-time RT-PCR and were normalized to β-actin levels. Cyclopamine treatment (10 μM) reduced the expression of *Gli-1* and *Bcl-2* at the mRNA level. The bars in the graph represent means±SEM (n=6/group). One-way ANOVA revealed significant difference. * *P*<0.05, ** *P*<0.01 versus tomatidine group.

We confirmed the involvement of mitochondria in cyclopamine-induced apoptosis by observing cleavage of caspase 9 (Figure 4A). Bcl family proteins have been shown to regulate the opening of mitochondrial permeability transition pores or the activity of Apaf-1. So we checked the protein level of Bcl-2 by Western blotting. We observed that the protein level of Bcl-2 was decreased in cyclopamine-treated cells, but not in tomatidine-treated cells (Figure 4A). Moreover, we also examined the mRNA level of *Bcl-2* using real-time RT-PCR. The treatment with cyclopamine reduced the expression of *Bcl-2* at the mRNA level (Figure 4B). The level of Gli-1, which indicates the activity of Hh signaling, was well correlated with the changes in that of Bcl-2 (Figure 4).

## 3. Discussion

Hh signaling is thought to play a role in the proliferation of gastric cancer cells. However, how it regulates the proliferation of gastric cancer is poorly characterized. In the present study, we show that Hh signaling regulates the survival of gastric cancer cells by regulating the expression of Bcl-2.

Apoptosis is the ubiquitous and highly regulated mechanism by which cells undergo programmed death [1]. Apoptosis plays an important role in many different stages of development and normal physiology. Moreover, during carcinogenesis, most cancer cells acquire the ability to overcome normal apoptotic processes. In particular, chemo-resistant cancer cells are resistant to apoptotic stimuli. Therefore, one of the important approaches for cancer therapy is the induction of apoptosis of cancer cells. Several signaling pathways, such as Fas/FasL, TNFα/TNF receptor, TRAIL/TRAIL-R, and EGF/EGFR, have been suggested as therapeutic targets to induce apoptosis in gastrointestinal tract cancers. The present study shows that Hh signaling can regulate apoptosis of gastric cancer cells and can be a good therapeutic target in gastric cancer.

The mechanisms that trigger apoptosis are often categorized into two main pathways – the extrinsic and intrinsic pathways. The extrinsic pathway (also known as the death receptor pathway) is the mechanism by which cells of the immune system trigger apoptosis in ‘unhealthy’ cells through ligand-mediated activation of cell surface death-mediating receptors [14–16], such as CD95/Fas/Apo1, TNF Receptor 1 (TNFR1), TNF Receptor 2 (TNFR2), and Death Receptors 3–6 (DR3-6) [1]. Ligand binding induces receptor multimerization, binding of the FADD adapter protein, formation of the death-induced signaling complex (DISC) with recruitment of the initiator caspases 8 and 10, and activation of the effector caspases 3 and 7 [14,16]. Cleavage of multiple cytoplasmic and nuclear substrates irreversibly culminates in cell destruction. The intrinsic pathway (also known as the mitochondrial pathway) can be triggered by any stimuli that cause oxidative stress, mitochondrial disturbances, and DNA damage, such as cancer therapeutic agents, hypoxia, ischemia-reperfusion injury and ionizing irradiation. These stimuli cause permeabilization of the outer mitochondrial membrane, which facilitates cytochrome c or Smac/Diablo release into the cytoplasm. The released cytochrome c then binds the caspase adaptor Apaf-1 (apoptotic protease-activating factor-1) thereby triggering the apoptotic cascade by activating pro-caspase 9 and forming a complex termed the apoptosome. This complex in turn activates several downstream effector caspases, such as caspase 3, 6, and 7, leading to DNA fragmentation and cell death [14,17–20]. In the present study, we present evidence suggesting that cyclopamine-induced apoptosis involves the intrinsic pathway. The first evidence is that cytochrome c was released from the mitochondria after cyclopamine treatment, as shown by double staining for mitotracker and cytochrome c (Figure 3). The second evidence in the present study is that caspase 9 was activated after cyclopamine treatment (Figure 4).

Bcl-2 (B-cell members of pro-apoptotic proteins) family is one group of key players in the mitochondrial pathway. It consists of more than 20 members of pro-apoptotic proteins (Bax, Bak, Bok, Bad, Bid, Bik, Bim, Bcl-Xs, Krk, Mtd, Nip3, Nix, Noxa and Bcl-B), and anti-apoptotic proteins (Bcl-2, Bcl-Xl, Mcl-1, Bfl-1, Bcl-W, and Bcl-G)[21–24]. Pro-apoptotic members of the Bcl-2 family trigger mitochondrial outer membrane permeabilization with release of proteins from the mitochondrial intermembrane space into the cytosol, including cytochrome c and Smac/Diablo, whereas anti-apoptotic members can bind and inactivate Apaf-1. In the present study, we show that Bcl-2 is involved in cyclopamine-induced apoptosis in gastric cancer cells (Figure 4). The regulation of Bcl-2 by Hh signaling has been shown in several other cancer cells. In medulloblastoma cells, increased expression of the Hh signaling target positively regulated Bcl-2 transcription, whereas pharmacological suppression of Hh activity resulted in decreased Bcl-2 expression and increased apoptosis [6,25]. Also, Hh signaling directly activates Bcl-2 expression by binding to a short region of the Bcl-2 promoter [26,27]. Basal cell carcinoma cells consistently express high levels of the anti-apoptotic Bcl-2 protein [28,29], while transgenic animal models have demonstrated that both Bcl-2 and Shh pathway members can contribute to multi-step skin carcinogenesis *in vivo* [30]. Hh signaling is able to positively regulate Bcl-2 transcription in primary keratinocytes in a dose-dependent manner [26]. Hh signaling is also able to induce endogenous Bcl-2 expression in Hh signaling-inducible transgenic mice.

## 4. Experimental Section

### 4.1. Cell Line

Human gastric cancer SNU16 cells were maintained as monolayer cultures in RPMI 1640 (Gibco, BRL) medium supplemented with 10% fetal bovine serum (FBS) (Life Technologies), 100 U/mL penicillin, and 100 μg/mL streptomycin and incubated at 37°C in a humidified atmosphere containing 5% CO_2_ in air. Cells were treated with ethanol (vehicle), tomatidine or cyclopamine at the indicated times and doses.

### 4.2. Proliferation Assays

SNU16 cells (1 × 10^3^ cells/well) were plated in 96-well flat-bottomed tissue-culture plates for 48 hrs at 37°C. For the experiment of Hh signal inhibition, the following day the medium was changed to RPMI with 0.5 % FBS, according to a previous report [6]. An inhibitor of the Hh signal pathway, cyclopamine [12,13] (Sigma-Aldrich), or its non-functional analog tomatidine (Sigma-Aldrich) added at a concentration of 10 μM. After incubation, 10 μL/well Cell Proliferation Reagent WST-1(Roche, Germany) were added and incubated for 4 hrs. Viable cell mass was determined by optical density measurements at 450 nm (OD450) at 2 and 4 days using the ELISA reader. Relative growth was calculated as OD (day 4) – OD (day 2)/OD (day 2).

### 4.3. Flow Cytometry

SNU16 cells (1 × 10^7^) in PBS supplemented with 0.5% BSA and 0.01% sodium azide were adjusted to 1 × 10^6^ cells/100 μL and used for flow cytometry. A total of 10,000 events were analyzed for each sample with a FACS Calibur (Becton Dickinson) using Cell-Quest software for collecting the samples and ModFit LT for determining the rates of different cell cycle and apoptotic cells.

### 4.4. Nuclear Morphology and Immunocytochemistry

Cells were fixed in 4% paraformaldehyde for 20 min at 4°C and processed for immunocytochemistry. After permeabilization with 0.1% saponin, the cultures were incubated overnight at 4°C with the rabbit polyclonal antibody anti-cytochrome c (sc-7159, Santa Cruz Biotechnology, Santa Cruz, CA) followed by incubation with a secondary donkey anti-rabbit Alexa Fluor 488 (Molecular Probes, Eugene, OR) for 1 hour at room temperature. To study the co-localization, mitochondria were labeled by incubation of cells with 200 nM Mitotracker Red (Molecular Probes) for 30 min at 37°C. The samples were fixed in 4% neutral buffered formaldehyde and mounted in Vectashield Mounting Media supplemented with 4′,6′-diamidino-2-phenylindole (DAPI, 5 mg/mL, Vector Laboratories, Burlingame, CA). The nuclei were evaluated using confocal microscope (Confocal, Olympus, Tokyo, Japan).

### 4.5. Western Blot

SNU16 cells (2 × 10^6^) were incubated for indicated hours at 37°C in RPMI 1640 medium containing 10% FBS. Then, the cells were collected and rinsed twice in PBS. The cell pellets were lysed for 1 hour in lysis buffer (50 mM Tris, pH 7.4, 150 mM NaCl, 2 mM EDTA, 2 mM EGTA, 0.3% NP-40, 100 μM PMSF, 10 μg/mL leupeptin, 2 μg/mL aprotinin) on ice and centrifuged at 12,000 rpm for 20 min at 4°C. Protein concentration was determined by standard Bradford method. Fifty μg of total protein were separated by SDS-PAGE using 12% polyacrylamide gel. The protein in gel was transferred to 0.45-μm nitrocellulose membrane with Trans-Blot SD Semi-Dry Electrophoretic Transfer Cell. The membranes were probed with each antibody. Mouse monoclonal anti-β-actin (C-2), rabbit anti-Bcl-2 polyclonal antibody (Santa Cruz Biotechnology, Inc., USA), rabbit anti-Gli-1 polyclonal antibody (Santa Cruz Biotechnology, Inc., USA) and rabbit anti-caspase 9 polyclonal antibody (Cell Signaling Technology, Inc., USA) were purchased. The blots were developed by a standard enhanced chemiluminescence (ECL) method (Amersham).

### 4.6. Real-Time RT-PCR

Total RNA was extracted from cells using RNeasy mini kit (Quiagen, Hilden, Germany). Total RNA from each sample (1 μg) was reverse transcribed with oligo(dT) 15 and random hexamer primers using M-MuLV reverse transcriptase (Promega, Madison, WI, USA). *Gli-1* and *Bcl-2* mRNA expression was analyzed by real-time RT-PCR using SYBR Green dye. In brief, 10 ng cDNA and gene-specific primers were added to SYBR Green PCR Master Mix (SYBR Green 1 Dye, AmpliTaq DNA polymerase, dNTPs with dUTP and optimal buffer components; Roche, Mannheim, Germany) and subjected to PCR amplification (50 cycles at 95°C for 10 s, 58°C for 10 s, 60°C for 10 s) in a Roche LightCycler. The amplified transcripts were quantified by the comparative method using β-actin as normalizing control as described previously [31]. The primer sequences are as follows: Gli-1, GCCGTGCTAAAGCTCCAGTGA and CTGCCCTATGTGAAGCCCTATTTG, Bcl-2, AACTGGGG GAGGATTGTGGC and GATCCAGGTGTGCAGGTGCC.

### 4.7. Data Analysis

The data are presented as means±SEM. The differences between the mean values of two groups were evaluated using the Student’s t-test (unpaired comparison). For comparison of more than three groups, we used one-way analysis of variance (ANOVA) test followed by Tukey’s multiple comparison. *P* values of <0.05 were considered statistically significant.

## 5. Conclusions

The present study suggests that Hh signaling regulates the survival of gastric cancer cells by regulating the expression of Bcl-2.
